# End-diastolic forward flow in repaired tetralogy of Fallot: Mid-term outcomes from a single center

**DOI:** 10.3389/fcvm.2022.1068752

**Published:** 2023-01-09

**Authors:** Ying Huang, Xiaowei Cai, Lishan Zhong, Wen Xie, Qi Lou, Jianrui Ma, Jimei Chen, Jian Zhuang, Shusheng Wen, Junfei Zhao

**Affiliations:** ^1^Department of Cardiovascular Surgery, Guangdong Cardiovascular Institute, Guangdong Provincial People’s Hospital, Guangdong Academy of Medical Sciences, Guangzhou, China; ^2^Department of Thoracic and Cardiovascular Surgery, Zhongnan Hospital of Wuhan University, Wuhan, China

**Keywords:** end-diastolic forward flow, restrictive physiology, risk factors, tetralogy of Fallot, transannular patch

## Abstract

**Background:**

Pulmonary arterial end-diastolic forward flow (EDFF) following repaired tetralogy of Fallot (rTOF) is recognized as right ventricular (RV) restrictive physiology, which is closely related to poor prognosis. This study sought to review mid-term experience and investigate the risk factors of EDFF in the rTOF patients.

**Methods:**

From September 2016 to January 2019, 100 patients (age < 18 years old) who underwent complete tetralogy of Fallot (TOF) repair were enrolled and were divided into EDFF group (*n* = 52) and non-EDFF group (*n* = 48) based on the presence of postoperative EDFF. Elastic net analysis was performed for variable selection. Univariate and multivariate logistic analyses were used to analyze the correlation between risk factors and EDFF.

**Results:**

End-diastolic forward flow group had lower systolic blood pressure (*P* = 0.037), diastolic blood pressure (*P* = 0.027), and higher vasoactive-inotrope score within 24 h after surgery (*P* = 0.022) than non-EDFF group. Transannular patch (TAP) was an independent predictor of postoperative EDFF [*P* = 0.029, OR: 2.585 (1.102∼6.061)]. Patients were followed up for a median of 2.6 years [interquartile range (IQR) 1.6] after the first TOF repair. During follow-up, the prevalence of the EDFF was lower in those with pulmonary valve (PV) reconstructions than that in those undergoing patch enlargement without PV reconstructions in the primary TOF repair (*P* < 0.001).

**Conclusion:**

End-diastolic forward flow was associated with TAP. Patients with EDFF might have a transient hemodynamic instability in the early postoperative period. PV reconstructions in the TOF repair might reduce the incidence of EDFF in the mid-term follow-up.

## 1. Introduction

Tetralogy of Fallot (TOF) is the most common type of cyanotic congenital heart disease (1). Although advanced surgical techniques have been applied to this complex cardiac anomaly, patients with repaired TOF (rTOF) can develop right ventricular (RV) diastolic dysfunction after surgery (2). RV restrictive physiology, referred to as abnormalities in RV diastolic function, has been observed both transiently at the time of initial repair and chronically at late follow-up in some rTOF patients (3, 4), which is closely related to poor prognosis (3, 5, 6).

End-diastolic forward flow (EDFF) in the main pulmonary artery, resulting from diastolic RV stiffness (4), is regarded as a manifestation of RV restrictive physiology in many studies (2, 7, 8). More and more attention has been paid to this phenomenon in rTOF patients (8, 9), whereas the risk factors of EDFF are not clear, which mostly focus on preoperative physiological state and surgical procedures, such as transannular patch (TAP) (6). Studies of the association between TAP and EDFF have yielded divergent results. Some authors have suggested that postoperative EDFF was associated with greater intraoperative myocardial injury and TAP (5, 6, 10). Others, in contrast, have found that TAP were not related to the EDFF (3, 11). The purpose of the present study was to report our mid-term experience and investigate the risk factors of EDFF in the rTOF patients.

## 2. Materials and methods

### 2.1. Study design and participants

This retrospective observational study enrolled 100 consecutive patients (age < 18 years old) who underwent complete TOF repair with cardiopulmonary bypass (CPB) from September 2016 through January 2019 at Guangdong Provincial People’s Hospital. Patients with pulmonary atresia, absent pulmonary valve, atrioventricular septal defect or double-outlet right ventricle, and without digital echocardiographic images were excluded. The included patients were divided into two groups based on the presence of EDFF in postoperative echocardiography or not. We retrospectively reviewed data that had been prospectively collected, including demographic data, hospital records, surgical records, and follow-up files. The study was conducted in accordance with the Declaration of Helsinki (as revised in 2013). The study was approved by the Ethics Committee of Guangdong Provincial People’s Hospital (No. GDREC 2018315H). Written informed consent was obtained from all subjects.

### 2.2. Surgical procedures

The surgical repair of TOF was all performed by complete median sternotomy with moderate hypothermic CPB. The approach for repair of right ventricular outflow tract (RVOT) was transatrial/transpulmonary (28/100, 28%), transventricular (54/100, 54%) and transatrial plus transventricular (18/100, 18%) incision. 63 (63/100, 63%) patients underwent TAP repair, of whom 15 underwent pulmonary valve (PV) reconstructions. Among patients with PV reconstructions, 13 underwent monocusp valve reconstructions and 2 underwent bicuspid valve reconstructions using expanded polytetrafluoroethylene (ePTFE) membrane. 37 (37/100, 37%) only underwent pulmonary valvotomy or hegar dilatation (Table 1). Systolic blood pressure (SBP) and diastolic blood pressure (DBP) were recorded at the end of CPB.

**TABLE 1:** Baseline and selected perioperative variables.

Characteristic	Non-EDFF (*n* = 48)	EDFF (*n* = 52)	*P-*value
Baseline variables			
Age at surgery, months	7 (3)	9 (8)	0.092
Age group at surgery, %			0.045
Younger infant (<0.5 year)	10 (20.8)	5 (9.6)	
Older infant (0.5–1 year)	27 (56.3)	27 (51.9)	
Toddler and preschool (2–5 year)	10 (20.8)	18 (34.6)	
School-aged and adolescent (6–18 year)	1 (2.1)	2 (3.8)	
Male, %	29 (60.4)	36 (69.2)	0.356
BSA, m^2^	0.38 (0.06)	0.40 (0.08)	0.097
SBP, mmHg	94 (13)	94 (15)	0.931
DBP, mmHg	54 (16)	53 (18)	0.879
SpO_2_, %	85 (17)	85 (15)	0.769
Preoperative variables			
A at tricuspid valve, cm/s	64 (31)	69 (25)	0.064
E/A at tricuspid valve	0.96 (0.60)	0.87 (0.47)	0.183
Indexed RVOT diameter, mm/m^2^	17.0 (5.2)	16.3 (5.5)	0.105
Indexed RPA diameter, mm/m^2^	19.5 (7.0)	17.7 (6.6)	0.101
Intraoperative variables			
TAP, %	25 (52.0)	38 (73.0)	0.030
PV treatments, %			0.078
Valvotomy or dilatation	23 (47.9)	14 (26.9)	
TAP with PV reconstruction	7 (14.6)	8 (15.4)	
TAP without PV reconstruction	18 (37.5)	30 (57.7)	
Repair approach, %			0.091
Transatrial/transpulmonary	18 (37.5)	10 (19.2)	
Transventricular	21 (43.8)	33 (64.5)	
Transatrial plus transventricular	9 (18.8)	9 (17.3)	
Postoperative variables			
Postoperative SBP, mmHg	90 (60)	83 (16)	0.037
Postoperative DBP, mmHg	56 (18)	48 (15)	0.027
VIS within 24 h	5 (5)	10 (5)	0.022

Continuous variables are expressed as median (IQR), categorical variables as percentage. A, A-wave velocity at the tricuspid valve; BSA, body surface area; DBP, diastolic blood pressure; EDFF, end-diastolic forward flow; PV, pulmonary valve; RPA, right pulmonary artery; RVOT, right ventricular outflow tract; SBP, systolic blood pressure; SpO_2_, pulse oxygen saturation; TAP, transannular patch; VIS, vasoactive-inotrope score.

### 2.3. Measurements and definitions

**FIGURE 1:**
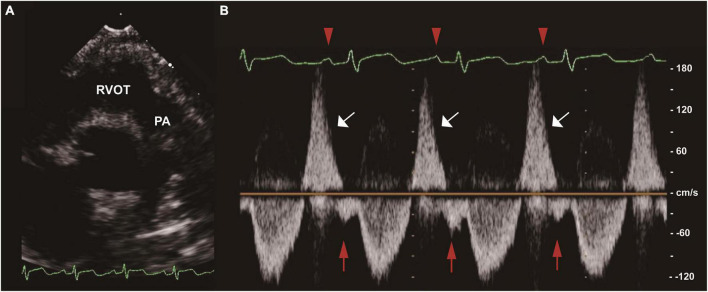
End-diastolic forward flow (EDFF) in a patient after tetralogy of Fallot (TOF) repair. **(A)** Two-dimensional image of the RVOT and PA in the parasternal long axis view. **(B)** Pulse wave Doppler of the main PA showing pulmonary regurgitation (white arrow) and late diastolic pulmonary artery forward flow (red arrow), a low velocity forward flow immediately following the p wave of the electrocardiogram (red arrowhead) and preceding the systolic forward flow. EDFF, end-diastolic forward flow; PA, pulmonary artery; RVOT, right ventricular outflow tract; TOF, tetralogy of Fallot.

Two-dimensional, M-mode and Doppler echocardiography were performed according to standard American Society of Echocardiography (ASE) guidelines (12, 13). The severity of PR was graded as normal, mild, moderate, and severe based on standard assessment by comprehensive echocardiogram (14). To simplify the analysis, the aforementioned six grades were combined into normal, mild, moderate and severe. Offline measurements of pulmonary artery pulsewave Doppler (parasternal long axis) were performed in all patients by an experienced sonographer before discharge (median time since surgery 7 days [IQR 5]), who had no prior knowledge of the study participants. In order to mitigate the effect of respirophasic variation, pulmonary arterial EDFF was considered to be present if identified in 3 consecutive cardiac cycles (Figure 1). All data in units of length or area were indexed to body surface area (BSA). Vasoactive-inotrope score (VIS) was calculated daily as per Gaies et al. (15), where VIS = dopamine dose (μg/kg/minute) + dobutamine dose (μg/kg/minute) + [100 × epinephrine dose (μg/kg/minute)] + [10 × milrinone dose (μg/kg/minute)] + [10000 × vasopressin dose (units/kg/minute)] + [100 × norepinephrine dose (μg/kg/minute)].

### 2.4. Follow-up

All patients who had been discharged alive were followed up 3, 6 months after surgery and at 1-year intervals thereafter to obtain echocardiographic and electrocardiographic indices. For patients using other centers for follow-up, we contacted their parents or guardians to obtain their health status *via* phone and WeChat.

### 2.5. Statistics

Data were presented as frequencies (percentages) for categorical variables and medians [interquartile range (IQR)] for continuous variables. Differences between groups were assessed using the 2 test or Fisher exact test for categorical variables and the Student *t*-test or the Mann–Whitney U test for continuous variables according to the data distribution. The elastic net analysis in a penalized logistic regression model (R package *glmnet*) was used to select the most useful prediction variables from all pre- and intraoperative candidates. Univariate logistic regression analysis and multivariate logistic stepwise regression analysis were used for risk factor analysis. Two-sided *P*-values of less than 0.05 were considered statistically significant. All analyses were performed using IBM SPSS Statistic version 25 (IBM Corp.) and R version 4.1.0.

## 3. Results

### 3.1. Baseline characteristics

Among the 100 patients that met the inclusion criteria for the study, the median age at the time of complete TOF repair was 8 months (IQR 7), and 65 (65%) were boys. There were 52 patients with postoperative EDFF. Group by EDFF did not reveal significant differences in baseline characteristics including age at operation, gender, BSA, preoperative blood pressure and pulse oxygen saturation (SpO2) (Table 1). Patients in the higher age group seemed to have a higher incidence of EDFF after being divided into four age groups (*P* = 0.045). In the 2 – 5 age group, patients with postoperative EDFF took a higher proportion, while a lower proportion in the <0.5 age group.

### 3.2. Operation details and early outcome

**FIGURE 2:**
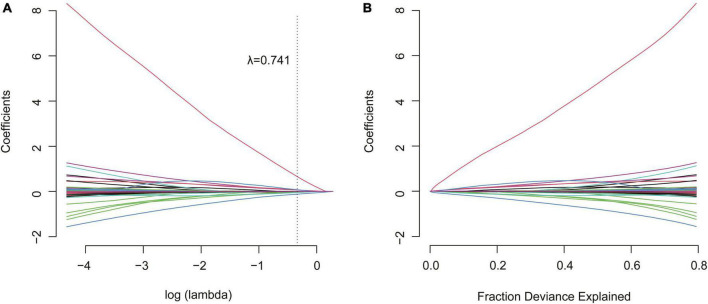
Elastic net model profile plots. **(A)** Coefficient profile plots showing how size of the coefficients of baseline, preoperative and intraoperative factors shrinks with increasing value of the λ penalty. Factors and their regression coefficients could be selected for the model when it attained to the optimal combination of α and λ for the elastic net model. Each curve represented one factors and there were 13 factors were obtained when the value of λ was 0.741. **(B)** The size of coefficients of preoperative and intraoperative factors increases with increasing value of the fraction deviance explained. The range of fraction deviance for the variation of the value of λ with variable selection was showed in the elastic net model.

The presence of EDFF appeared more prevalent in those who underwent TAP repair (*P* = 0.030). The different approaches of RVOT repair (*P* = 0.091) and PV treatments (*P* = 0.078) showed no significant difference between the EDFF and non-EDFF group in the early postoperative period. In total, 4 preoperative factors and 7 intraoperative factors and their regressions coefficients were selected for the model based on the optimal λ (0.741) for elastic net model, all of them were identified to be associated with postoperative EDFF (Figure 2 and Table 1). Then they were included in the univariate logistic regression analysis, in which those with *P* < 0.1 were further included in the multivariate logistic stepwise regression analysis. TAP was an independent predictor of EDFF [*P* = 0.029, OR: 2.585 (1.102∼6.061)] (Table 2).

**TABLE 2:** Multivariate predictors of end-diastolic forward flow (EDFF).

Characteristic	Univariate analysis		Multivariate analysis	
	**OR (95% CI)**	***P-*value**	**OR (95% CI)**	***P-*value**
A	1.020 (0.999∼1.042)	0.064		
E/A	0.436 (0.186∼1.019)	0.055	0.427 (0.182∼1.005)	0.051
RVOT diameter	0.916 (0.830∼1.010)	0.078		
RPA diameter	0.923 (0.841∼1.012)	0.089		
TAP	2.497 (1.094∼5.752)	0.032	2.585 (1.102∼6.061)	0.029
Valvotomy or dilatation	0.400 (0.174∼0.922)	0.032		
TAP without PV reconstruction	2.273 (1.019∼5.071)	0.045		
Transatrial/Transpulmonary	0.397 (0.161∼0.980)	0.045		
Transventricular	2.233 (1.001∼4.982)	0.050		

A, A-wave velocity at the tricuspid valve; EDFF, end-diastolic forward flow; E/A, ratio between early (E) and late atrial (A) ventricular filling velocity; OR, odds ratio; RPA, right pulmonary artery; RVOT, right ventricular outflow tract; TAP, transannular patch.

During postoperative hospitalization, one patient underwent diaphragmatic plication due to diaphragmatic paralysis, and one patient underwent skin lesion resection due to incision infection. There was no early in-hospital death. Though no significant difference was observed between the two groups in postoperative hospital stay (*P* = 0.254), ICU stay (*P* = 0.261) or ventilation duration (*P* = 0.227), patients with EDFF had lower postoperative SBP (*P* = 0.037), DBP (*P* = 0.027), and higher VIS within 24 h after surgery [10 (IQR 5) vs 5 (IQR 5), *P* = 0.022], compared with patients without EDFF (Table 1).

### 3.3. Follow-up

Patients were followed up for a median of 2.6 years [IQR (1.6)] after the first TOF repair with 3 lost to follow-up randomly. Among these three patients lost to follow-up, one had postoperative EDFF and the other two didn’t appear EDFF in the early postoperative period. Among the remaining patients, there was no late death. During the follow-up, reoperations were performed in four patients, of whom one had postoperative EDFF and the other three didn’t appear EDFF in the early postoperative period. No statistically significant difference was observed in reintervention rate between EDFF and non-EDFF group (*P* = 0.348). In non-EDFF group, one patient underwent percutaneous balloon pulmonary valvuloplasty within a year of discharge, because of the pulmonary stenosis resulted from pulmonary valve reconstruction in the primary repair. One patient underwent RVOT patches enlargement at the fifth year after discharge, due to inadequate excision of subvalvular muscle bundle in the primary repair with the PV dilatation. One patient underwent reoperation for distal left pulmonary stenosis. In the EDFF group, the patient underwent RVOT patches enlargement at the fourth year after discharge, due to inadequate excision of subvalvular muscle bundle in the primary repair with the PV dilatation. There was no statistically significant difference at the operative age between patients with and without re-intervention (*P* = 0.632).

**FIGURE 3:**
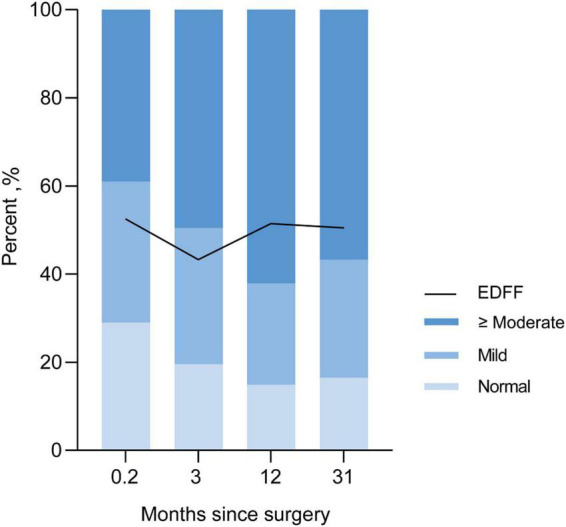
Incidence of EDFF and rate of different severity of pulmonary regurgitation in postoperative and follow-up echocardiography. EDFF, end-diastolic forward flow.

**FIGURE 4:**
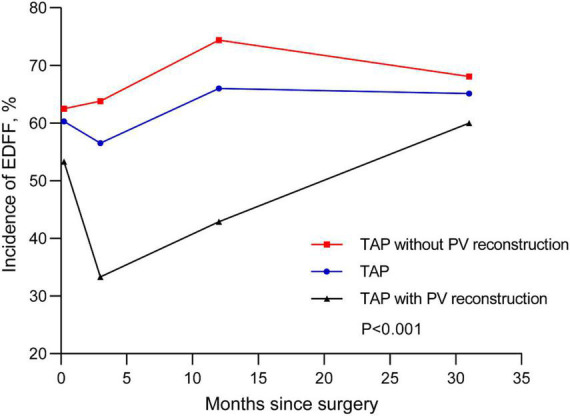
End-diastolic forward flow in postoperative and follow-up echocardiography for patients with TAP. The lines are shown for temporal trend of EDFF in patients with TAP and two subsets, including TAP with and without PV reconstructions. EDFF, end-diastolic forward flow; PV, pulmonary valve; TAP, transannular patch.

We recorded three follow-ups with a median time of 3, 12, and 31 months, respectively. The incidence of EDFF was 52, 43.3, 52.1, and 49.5% at discharge, the fist, the second, and the last follow-up, respectively. The rate of pulmonary regurgitation (PR) above moderate degree was 39, 49.5, 62.2, and 56.7% in above four periods, respectively (Figure 3 and Table 3). Patients with PV dilatation had a lower incidence of postoperative EDFF than surgically corrected patients during follow-up (*P* < 0.001). For patients with TAP, the incidence of EDFF was 60.3, 56.5, 66.0, and 66.1% at discharge, the fist, the second, and the last follow-up, respectively. Among the patients with TAP, the incidence of EDFF in those without PV reconstruction was 62.5, 63.8, 74.4, and 68.1% at above four periods, respectively (Figure 4). For patients with monocusp valve reconstructions, the incidence of EDFF was 53.8, 30.8, 33.3, and 61.5% at above four periods, respectively. For two patients with bicuspid valve reconstructions, one always had EDFF from the postoperative to follow-up period and another one only appeared EDFF at the second follow-up.

**TABLE 3:** Postoperative and follow-up echocardiography.

Characteristic	Non-EDFF	EDFF	*P*-value
Postoperative			
Time since surgery, days	8 (6)	7 (6)	0.128
Number of cases, %	48 (48.0)	52 (52.0)	
PR degree, %			0.151
Normal	16 (33.3)	13 (25.0)	
Mild	17 (35.4)	15 (28.8)	
≥Moderate	15 (31.3)	24 (46.2)	
First follow-up			
Time since surgery, months	3 (1)	3 (1)	0.622
Number of cases, %	55 (56.7)	42 (43.3)	
PR degree, %			<0.001
Normal	17 (30.9)	2 (4.8)	
Mild	22 (40.0)	8 (19.0)	
≥Moderate	16 (29.0)	32 (76.2)	
Last follow-up			
Time since surgery, months	30 (29)	32 (19)	0.184
Number of cases, %	49 (50.5)	48 (49.5)	
PR degree, %			<0.001
Normal	15 (30.6)	1 (2.1)	
Mild	20 (40.8)	6 (12.5)	
≥Moderate	14 (28.6)	41 (85.4)	

Continuous variables are expressed as median (IQR), categorical variables as percentage. EDFF, end-diastolic forward flow; PR, pulmonary regurgitation.

Both the two follow-up echocardiography showed significant differences in the severity of PR between the EDFF and non-EDFF group (*P* < 0.001, *P* < 0.001, respectively), while the postoperative echocardiography showed no significant difference (*P* = 0.151) (Table 3). Moreover, between PV reconstruction and non-PV reconstruction groups, significant differences were observed in the severity of PR in the postoperative and first follow-up echocardiography (*P* < 0.001, *P* = 0.034, respectively), but not in the last follow-up echocardiography (*P* = 0.114).

## 4. Discussion

Patients with EDFF might be exposed to a transient hemodynamic instability in the early postoperative period. TAP tended to predict a higher prevalence of EDFF. During follow-up, EDFF was closely related to the severity of PR, while PV reconstructions in the TOF repair might reduce the incidence of EDFF and alleviate the PR.

The median age at operation of our patients was 8 months (IQR 7). It was recommended that patients should undergo the complete repair of TOF by age 6 months (but no later than 12 months) (16), since older age was reported to be associated with a higher rate of in-hospital complications (17). In our center, the incidence of EDFF seemed to be higher in the higher age group. It seemed that early operation under 6 months could prevent postoperative EDFF, and the incidence of EDFF might increase in patients undergoing complete repair over than 1 year old, which might be attributed to a stiffer right ventricle and higher diastolic dysfunction due to prolonged cyanosis (18). In early studies (3, 5), the presence of EDFF was related to poor prognosis in terms of prolonged ICU stay and low cardiac output state in rTOF patients. As the standardization of surgical procedures, the progress of CPB and better postoperative management strategy have improved the early prognosis of TOF patients (16), significant differences were not observed between the EDFF and non-EDFF groups in postoperative hospital stay, ICU stay or ventilation duration in our patients. However, it was observed that the lusitropic response of the RV to β adrenergic agents was abnormal after surgery in rTOF patients (19). And another study of fifty rTOF patients with a median of age at 5 years reported that postoperative EDFF was correlated to a longer duration of inotropic support (6). Similarly, we found that rTOF patients with EDFF had higher VIS within 24 h after surgery, accompanied by lower SBP, DBP after CPB. It has been confirmed that VIS calculated in the first 24 h after cardiac ICU admission was strongly associated with outcomes in infants undergoing cardiac surgery (15), and used as an important indicator to evaluate the outcome of rTOF patients (17). Therefore, a transient hemodynamic instability might have occurred in the early postoperative period in such patients, which might need more vasoactive drugs to maintain the blood pressure.

The prevalence of EDFF among rTOF patients was 52, 43.3, and 49.5% at perioperative period, the first follow-up with a median time of 3 months and the last follow-up with a median time of 31 months, respectively. A recent systematic review and meta-analysis demostrated that the prevalence of EDFF among patients with repaired TOF was 46.5% (95% CI, 41.6–51.3%), which was closed to the result of our center (9). In our cohort, patients with PV dilatation had a lower incidence of EDFF than surgically corrected patients, and patients with TAP repair were more likely to appear EDFF after surgery. EDFF was typically associated with TAP and not usually present in rTOF patients in whom the pulmonary valve had been preserved during primary repair (20, 21).

Transannular patch is selected based on the anatomy and hemodynamics of RVOT in the complete TOF repair and usually represents a larger RVOT incision, destruction of PV, and more severe PR after surgery (22). Therefore, the fundamental reason for EDFF might be the above pathophysiological factors.

There was a view that transatrial/transpulmonary approach could protect right ventricular function (23–25). Considering edema from RVOT ventriculotomy, which might resulted in myocardial injury (26), TAP tended to predict a higher prevalence of EDFF in the early postoperative period. No significant difference was observed between EDFF and non-EDFF groups in the severity of PR in this period, which might be attributed to PV reconstructions applied to some patients with TAP. It have been reported that monocusp or bicuspid valve reconstructions can prevent immediate PR (27, 28).

Our patients with EDFF had a more severe PR in the first follow-up with a median time of 3 months and the last follow-up with a median time of 31 months, respectively. Two cross-sectional studies of rTOF patients more than 10 years after surgery demostrated that EDFF was correlated to the severity of PR (7, 29), which was consistent with observations in the follow-up of our patients. It was proposed that more severe PR as a particular hemodynamic state might allow the presence of EDFF (7). The chronic RV volume overload derived from longstanding PR might change the regional diastolic RV myocardial mechanical characteristics (29), leading to larger RV volumes and RV fibrosis in the follow-up, which might result in RV restrictive physiology (30, 31).

Moreover, we found that in patients with TAP repair, the incidence of EDFF in those undergoing PV reconstructions was lower than that in those without PV reconstructions since the surgery. There have been researches reported that monocusp or bicuspid valve reconstructions could improve short-term clinical outcomes in rTOF patients (27, 28, 32), but few studies have reported the influence of PV reconstructions on EDFF in such patients. Similarly, in our study, patients with PV reconstructions showed less PR during the postoperative period and first follow-up. A number of researches have reported that monocusp or bicuspid valve of PTFE membrane could prevent short-term PR (27, 28, 33, 34), which might explain the lower incidence of EDFF in patients with PV reconstructions in our center. However, this difference didn’t persist to the last follow-up with a median time of 31 months. It showed the limited duration of artificial PV of PTFE membrane (33), which might be ascribed to the inconsistency between the growth of RVOT and the lack of growth potential in prosthetic valves (34). Furthermore, the results of the incidence of EDFF in cases of TAP, monocusp and bicuspid valve reconstructions showed that the time interval for deterioration of EDFF in cases of TAP seemed to be shorter than that in cases of PV reconstructions. Therefore, PV reconstruction might delay the deterioration of EDFF in this study though its duration was limited. A longer follow-up is necessary for these rTOF patients to observe the long-term prognosis.

## 5. Limitations

This study had several limitations. First, a multi-center and large-sample prospective study will be required to further verify the results of this study. Second, a longer follow-up is necessary for these rTOF patients to observe the long-term prognosis. Last, more variables could be included into this study to further investigate the risk factors of EDFF.

## 6. Conclusion

End-diastolic forward flow was associated with TAP. Patients with EDFF might have a transient hemodynamic instability and need more vasoactive drugs in the early postoperative period. PV reconstructions in the primary TOF repair might reduce the incidence of EDFF in the mid-term follow-up.

## Data availability statement

The raw data supporting the conclusions of this article will be made available by the authors, without undue reservation.

## Ethics statement

The studies involving human participants were reviewed and approved by the Ethics Committee of Guangdong Provincial People’s Hospital. Written informed consent to participate in this study was provided by the participants’ legal guardian/next of kin.

## Author contributions

YH, XC, and LZ conducted the data collection, statistical analysis, and wrote the manuscript. WX, QL, and JM conducted the data validation. JC and JZ provided the funding support and supervision. JFZ and SW revised the manuscript. All authors contributed to the article and approved the submitted version.
